# Evaluating the prognostic value of admission IL-8 and sST2 as biomarkers of early myocardial injury in severe community-acquired pneumonia

**DOI:** 10.1080/07853890.2026.2709277

**Published:** 2026-07-30

**Authors:** Shanshan Liu, Lu Liu, Jian Dong

**Affiliations:** ^a^ Neurocritical Care Unit, Union Jiangbei Hospital, Huazhong University of Science and Technology, Wuhan, Hubei Province, China; ^b^ Department of Respiratory and Critical Care Medicine, Union Jiangbei Hospital, Huazhong University of Science and Technology, Wuhan, Hubei Province, China

**Keywords:** Severe community-acquired pneumonia, myocardial injury, interleukin-8, soluble ST2, high-sensitivity cardiac troponin I, risk stratification

## Abstract

**Background:**

Myocardial injury in severe community-acquired pneumonia (SCAP) is often under-recognized, and interpretation of high-sensitivity cardiac troponin I (hs-cTnI) may be complicated by renal dysfunction and critical illness. We evaluated whether admission interleukin-8 (IL-8) and soluble suppression of tumorigenicity 2 (sST2) could support early risk stratification of guideline-consistent myocardial injury in patients with SCAP.

**Methods:**

In this prospective cohort of 146 patients with SCAP, guideline-consistent myocardial injury was defined as hs-cTnI elevation above the assay-specific 99th percentile upper reference limit. Admission IL-8, sST2, and their combination were assessed using receiver operating characteristic curve analysis and 10-fold cross-validation. Fixed and estimated glomerular filtration rate (eGFR)-stratified hs-cTnI thresholds were compared in an exploratory analysis. An exploratory subgroup analysis was performed among patients with CURB-65 ≤ 1.

**Results:**

The median age was 71 years; 91 patients (62.3%) were male and 55 (37.7%) were female. Myocardial injury occurred in 88 patients (60.3%). The IL-8 + sST2 model showed good discrimination for myocardial injury, with an AUC of 0.825 (95% CI: 0.751–0.895) and an internally cross-validated AUC of 0.812. Admission IL-8 showed stronger individual discrimination than sST2. eGFR-stratified hs-cTnI thresholds did not reduce discordant hs-cTnI elevations in this cohort, likely because few patients had moderate-to-severe renal dysfunction. Among patients with CURB-65 ≤ 1 (*n* = 38), IL-8 > 43.53 pg/mL was associated with a higher prevalence of myocardial injury (76.2% vs. 29.4%, *p* = 0.004). An exploratory 0–2 biomarker score showed a graded increase in myocardial injury prevalence across score categories: 15.4%, 62.5%, and 78.6%.

**Conclusions:**

Admission IL-8 was associated with guideline-consistent myocardial injury in patients with SCAP, whereas sST2 showed weaker individual discrimination. The combined model had numerically higher discrimination than IL-8 alone, but the incremental contribution of sST2 remains uncertain. These findings represent an initial evaluation and require external validation before clinical implementation.

## 1. Introduction

Community-acquired pneumonia (CAP) remains a major cause of infection-related hospitalization, organ dysfunction, and mortality worldwide. Among hospitalized patients, severe community-acquired pneumonia (SCAP) represents a high-risk clinical phenotype characterized not only by pulmonary infection and respiratory failure, but also by systemic inflammation, endothelial dysfunction, sepsis-related organ injury, and cardiovascular complications [1]. Current pneumonia severity tools, including CURB-65, the Pneumonia Severity Index (PSI), and the Sequential Organ Failure Assessment (SOFA) score, are widely used to estimate overall disease severity and mortality risk. However, these tools were not designed to identify early myocardial injury during the acute inflammatory phase of SCAP [2]. Furthermore, they do not specifically capture myocardial stress or inflammation-related cardiac involvement, which are central to SCAP-associated cardiovascular complications.

Myocardial injury is increasingly recognized as a clinically relevant complication of severe infection and acute respiratory illness. In SCAP, myocardial injury may result from overlapping mechanisms, including cytokine-mediated myocardial stress, hypoxemia, coronary microvascular dysfunction, endothelial activation, myocardial oxygen supply–demand imbalance, and sepsis-related myocardial depression [3,4]. These mechanisms may coexist with pre-existing cardiovascular disease or renal dysfunction, making early recognition difficult in routine clinical practice. Patients may develop myocardial injury without typical ischemic symptoms, and cardiac involvement may be overlooked when clinical attention is focused primarily on respiratory failure and infection control [5].

High-sensitivity cardiac troponin I (hs-cTnI) is central to contemporary definitions of myocardial injury. According to the Fourth Universal Definition of Myocardial Infarction, myocardial injury is identified when cardiac troponin values exceed the assay-specific 99th percentile upper reference limit, and acute myocardial injury is supported by a rise and/or fall pattern when serial measurements are available [6–8]. Nevertheless, interpretation of hs-cTnI in SCAP is complex. Troponin elevation may reflect acute myocardial injury, chronic myocardial injury, renal dysfunction, systemic critical illness, or type 2 myocardial infarction triggered by infection-related oxygen supply–demand mismatch [9,10]. In particular, renal dysfunction—common in SCAP—may elevate hs-cTnI independently of myocardial injury, further complicating interpretation [11,12]. Therefore, although hs-cTnI is essential for defining myocardial injury, additional biomarkers may help characterize inflammatory and myocardial stress pathways associated with cardiac involvement in SCAP [13,14].

This distinction is important for biomarker modelling. When hs-cTnI is used to define myocardial injury, it should not be treated as an independent predictor of the same outcome, because doing so may introduce circularity and inflate apparent diagnostic performance. Accordingly, the evaluation of non-troponin biomarkers may provide additional information on biological processes associated with myocardial injury while preserving a guideline-consistent outcome definition.

Interleukin-8 (IL-8) is a pro-inflammatory chemokine involved in neutrophil recruitment, endothelial activation, and amplification of systemic inflammatory responses. In severe infection, elevated IL-8 may reflect inflammatory burden and microvascular stress [15,16]. Soluble suppression of tumorigenicity 2 (sST2), a member of the interleukin-1 receptor family, is released in response to myocardial strain, inflammation, and tissue injury. Unlike troponin, which primarily reflects myocardial cell injury, sST2 may provide information on myocardial stress and inflammation-related cardiac remodelling pathways [17–19]. Thus, IL-8 and sST2 capture distinct but potentially complementary biological processes—IL-8 reflecting systemic inflammatory amplification and endothelial activation, and sST2 reflecting myocardial mechanical stress and fibrotic pathways—offering a dual-mechanism rationale for their combined evaluation in SCAP-associated myocardial injury [20,21].

In this study, we evaluated admission IL-8 and sST2 for early risk stratification of guideline-consistent myocardial injury in patients with SCAP. We hypothesized that admission IL-8 and sST2, alone or in combination, would be associated with guideline-consistent myocardial injury and provide risk information complementary to conventional severity scores. We assessed the discriminatory performance of IL-8, sST2, and a combined IL-8 + sST2 model, compared their performance with conventional severity scores, and developed an exploratory simplified biomarker score. We also examined, as a secondary exploratory analysis, whether eGFR-stratified hs-cTnI thresholds reduced discordant hs-cTnI elevations compared with a conventional fixed threshold [22]. Finally, we explored biomarker performance among patients classified as low risk by CURB-65 (≤1). All subgroup and threshold-adjustment analyses were pre-specified as exploratory. Because no external validation cohort was available, this study was designed as an initial evaluation of candidate biomarkers rather than as a definitive validation study.

## 2. Materials and methods

### 2.1. Study design and ethical approval

This was a single-center, prospective observational cohort study conducted at Union Jiangbei Hospital, Huazhong University of Science and Technology, between August 2024 and December 2025. The study protocol was approved by the institutional ethics committee of Union Jiangbei Hospital, Huazhong University of Science and Technology (approval number: LLHZ2024080101). Written informed consent was obtained from all participants or their legally authorized representatives before enrollment. The study was conducted in accordance with the Declaration of Helsinki and reported following the STROBE guidelines for observational studies[23].

### 2.2. Patient selection and eligibility criteria

Adult patients aged 18 years or older were screened if admitted with community-acquired pneumonia. CAP was diagnosed according to the 2019 IDSA/ATS guideline criteria, including acute respiratory symptoms, new pulmonary infiltrates on chest imaging, and exclusion of hospital-acquired pneumonia or alternative specific aetiologies [24].

SCAP was defined by at least one major criterion or three or more minor criteria. Major criteria included invasive mechanical ventilation or septic shock requiring vasopressors. Minor criteria included PaO_2_/FiO_2_ ≤250 mmHg, multilobar infiltrates, respiratory rate ≥30 breaths/min, altered mental status, blood urea nitrogen ≥20 mg/dL, leukopenia (<4 × 10^9^/L), thrombocytopenia (<100 × 10^9^/L), hypothermia (<36 °C), or hypotension requiring aggressive fluid resuscitation.

Exclusion criteria were age <18 years, pregnancy or lactation, terminal malignancy, death within 24 h of admission, declined consent, end-stage kidney disease requiring long-term dialysis, confirmed acute coronary syndrome, including ST-segment elevation myocardial infarction or non-ST-segment elevation myocardial infarction, or recent coronary intervention. Patients with missing key variables for outcome classification or primary biomarker analysis were also excluded. After data cleaning, 146 patients were included in the final analysis.

### 2.3. Clinical data collection

Clinical data were collected within 24 h of admission using a standardized form. Demographic variables included age, sex, body mass index, and smoking status. Comorbidities included chronic obstructive pulmonary disease, hypertension, diabetes mellitus, coronary artery disease, cerebrovascular disease, and chronic kidney disease, defined by documented medical history, medication use, or laboratory evidence consistent with each diagnosis before the acute admission. Baseline cardiovascular characteristics, including prior percutaneous coronary intervention or coronary artery bypass grafting and baseline antiplatelet therapy, were also recorded.

Hypertension was defined as a documented medical history, use of antihypertensive medication, or systolic blood pressure ≥140 mmHg and/or diastolic blood pressure ≥90 mmHg before admission. Diabetes mellitus was defined as a documented diagnosis, use of glucose-lowering medication, fasting glucose ≥7.0 mmol/L, or HbA1c ≥6.5%. Coronary artery disease was defined as a documented history of myocardial infarction, prior coronary revascularization, or angiographically confirmed coronary stenosis ≥50%.

Vital signs, respiratory parameters, routine laboratory tests, cardiac biomarkers, renal and liver function tests, coagulation markers, arterial blood gases, treatment variables, and in-hospital complications were recorded. Disease severity was quantified using the CURB-65 score for pneumonia severity assessment, the Pneumonia Severity Index (PSI) for estimating pneumonia-related mortality risk, and the Sequential Organ Failure Assessment (SOFA) score for evaluating the extent of organ dysfunction [25–27].

### 2.4. Biospecimen collection and laboratory analyses

Venous blood samples were obtained within 24 h of admission. Admission IL-8, sST2, hs-cTnI, interleukin-6, C-reactive protein, procalcitonin, B-type natriuretic peptide, D-dimer, complete blood count, liver function, renal function, and routine biochemical markers were measured.IL-8 was measured using the IL-8 Assay Kit (chemiluminescence immunoassay; Sichuan Wondfo Biotechnology Co., Ltd., China), with a detection range of 0–7500 pg/mL, an intra-assay coefficient of variation <8%, and an inter-assay coefficient of variation <10%. Serum sST2 concentrations were measured using a fluorescence immunoassay kit (Boditech Med Inc., Republic of Korea) according to the manufacturer’s instructions. hs-cTnI was measured using the Maccura i3000 fully automated chemiluminescence immunoassay platform (Maccura Biotechnology Co., Ltd., China). The local laboratory reference cutoff was 20 ng/L, and concentrations below 20 ng/L were reported quantitatively and retained as the measured values rather than being censored or imputed. For the study outcome classification, myocardial injury was defined using the assay-specific, sex-specific 99th-percentile upper reference limits provided by the manufacturer: 33.5 ng/L for males and 19.8 ng/L for females. The overall 99th-percentile upper reference limit was 26.4 ng/L and was used only in the prespecified exploratory analyses.

### 2.5. Primary outcome: guideline-consistent myocardial injury

The primary outcome was guideline-consistent myocardial injury, defined as an admission hs-cTnI concentration above the assay-specific, sex-specific 99th-percentile upper reference limit (33.5 ng/L for males and 19.8 ng/L for females), in accordance with the Fourth Universal Definition of Myocardial Infarction[8]. The primary classification was based on the hs-cTnI measurement obtained within 24 h of admission. When serial hs-cTnI measurements were available, a rise and/or fall pattern was considered supportive of acute myocardial injury but was not required for primary outcome classification. Because serial measurements and pre-admission baseline hs-cTnI values were unavailable for all patients, acute and chronic myocardial injury could not be reliably distinguished in every case. Therefore, the primary endpoint was myocardial injury rather than acute myocardial injury.

Patients with confirmed type 1 myocardial infarction were excluded. Type 2 myocardial infarction and non-ischemic myocardial injury were not systematically distinguished because complete serial hs-cTnI, electrocardiographic, and cardiac imaging data were unavailable for mechanistic adjudication in all patients. Evidence of myocardial ischemia was not required for the primary endpoint, which encompassed myocardial injury irrespective of its underlying mechanism.

### 2.6. Exploratory analysis: eGFR-stratified hs-cTnI interpretation

Because renal dysfunction may influence hs-cTnI concentrations, an exploratory analysis compared the manufacturer-provided overall 99th-percentile URL (26.4 ng/L) with eGFR-stratified hs-cTnI thresholds. The stratified thresholds were 26.4 ng/L for eGFR ≥60 mL/min/1.73 m^2^, 39.6 ng/L for eGFR 30–<60 mL/min/1.73 m^2^, and 66.0 ng/L for eGFR <30 mL/min/1.73 m^2^, using the proportional adjustment approach derived from Chen et al. [11].

These thresholds have been proposed to improve diagnostic specificity in patients with renal impairment, although they have not been independently validated in SCAP populations. The primary comparison was the number of discordant hs-cTnI elevations, defined as hs-cTnI elevation by a given threshold in patients not classified as having guideline-consistent myocardial injury based on sex-specific URLs in the final study dataset. Because only 18 patients had eGFR <60 mL/min/1.73 m^2^, including very few patients with moderate-to-severe renal dysfunction, the study lacked sufficient power to reliably estimate a continuous eGFR-by-hs-cTnI interaction. Therefore, formal interaction testing was not performed, and the eGFR-stratified hs-cTnI threshold analysis was considered descriptive and exploratory.

### 2.7. Exploratory subgroup analysis: CURB-65 ≤ 1 patients

An exploratory subgroup analysis was performed among patients classified as low risk by CURB-65 (score ≤1). The rationale was that traditional pneumonia severity scores may underestimate myocardial injury risk in patients without overt clinical severity.

Within this subgroup, patients were stratified using the overall cohort-derived ROC optimal cut-offs for IL-8 and sST2. The prevalence of myocardial injury was compared between biomarker-stratified groups. Interaction terms were included in logistic regression models to explore whether biomarker associations differed between patients with CURB-65 ≤ 1 and the remainder of the cohort. Because of the limited subgroup sample size, these analyses were considered exploratory.

### 2.8. Candidate biomarker model and simplified score

The primary candidate biomarkers were admission IL-8 and sST2. IL-8 was selected as a marker of systemic inflammatory activation, and sST2 was selected as a marker of myocardial stress and inflammation-related tissue injury. The primary model included IL-8 and sST2 only and excluded hs-cTnI because hs-cTnI contributed to the outcome definition.

To improve interpretability, an exploratory simplified score was constructed using ROC-derived optimal cut-offs. One point was assigned for IL-8 above the threshold and one point for sST2 above the threshold, yielding a total score of 0–2. The prevalence of myocardial injury was assessed across score categories.

### 2.9. Statistical analysis

Continuous variables were summarized as median and interquartile range, and categorical variables were summarized as number and percentage. Between-group comparisons used the Mann–Whitney U-test for continuous variables and the χ^2^ test or Fisher’s exact test for categorical variables.

Receiver operating characteristic curve analysis evaluated the discriminatory performance of IL-8, sST2, the combined IL-8 + sST2 model, and conventional severity scores. The area under the curve, 95% confidence interval, sensitivity, specificity, Youden index, and optimal cut-offs were calculated. Pairwise AUC comparisons were performed using the DeLong test with Bonferroni correction.

The combined model was constructed using admission IL-8 and sST2. Because both biomarkers had right-skewed distributions, log-transformed IL-8 and sST2 values were used for model construction. Other continuous variables were analysed using their original scales, and no additional normalization or mathematical transformations were applied before analysis. Internal performance was assessed using repeated 10-fold cross-validation, and the mean cross-validated AUC was reported. Univariable logistic regression examined associations with myocardial injury, reporting odds ratios per clinically interpretable unit: per 10 pg/mL for IL-8, per 10 ng/mL for sST2, per 10 mL/min/1.73 m^2^ for eGFR, per 1 point for SOFA and CURB-65, and per 10 points for PSI.

For the exploratory comparison of conventional versus eGFR-stratified hs-cTnI thresholds, descriptive statistics were reported. For the CURB-65 ≤ 1 subgroup, interaction terms were included in logistic regression models to evaluate whether biomarker associations differed between subgroups.

Missing data were assessed before analysis. No statistical imputation was performed. Patients with missing key variables required for outcome classification or primary biomarker modelling were excluded from the corresponding analyses, whereas variables with limited missingness were analysed using available-case analysis. Potential outliers were identified by inspection of source data and variable distribution plots. Extreme biomarker values were retained if they were biologically plausible and not attributable to data entry or measurement errors. No winsorization, truncation, or exclusion of outliers was performed.

All tests were two-sided, and *p* < 0.05 was considered statistically significant unless otherwise specified. Analyses were performed using SPSS version 27.0 and R version 4.3.0.

## 3. Results

### 3.1. Study population and baseline characteristics

A total of 146 patients with severe community-acquired pneumonia were included in the final analysis. According to the guideline-consistent definition, myocardial injury was identified in 88 patients (60.3%), whereas 58 patients (39.7%) were classified as having no myocardial injury.

Baseline characteristics according to myocardial injury status are summarized in Table 1. Patients with guideline-consistent myocardial injury had higher admission IL-8 and sST2 levels, higher hs-cTnI concentrations by definition, lower eGFR, lower PaO_2_/FiO_2_ ratios, and higher SOFA, CURB-65, and PSI scores. Respiratory failure and the requirement for non-invasive ventilatory support were also more frequent in the myocardial injury group. Age, sex distribution, and major baseline comorbidities were broadly similar between groups.

**Table 1.** Baseline characteristics according to guideline-consistent myocardial injury status.

Variable	Overall (*N* = 146)	Myocardial injury (*n* = 88)	Non-myocardial injury (*n* = 58)	P value
**Demographics**				
Age, years	71.00 (62.00–78.00)	72.00 (63.00–78.75)	70.00 (61.00–77.25)	0.682
Male sex	91/146 (62.3)	54/88 (61.4)	37/58 (63.8)	0.765
**Comorbidities**				
COPD	45/146 (30.8)	31/88 (35.2)	14/58 (24.1)	0.153
Hypertension	78/146 (53.4)	52/88 (59.1)	26/58 (44.8)	0.088
History of coronary artery disease, n (%)	27/146 (18.5)	18/88 (20.5)	9/58 (15.5)	0.449
Chronic kidney disease	5/146 (3.4)	4/88 (4.5)	1/58 (1.7)	0.650
Diabetes	24/146 (16.4)	17/88 (19.3)	7/58 (12.1)	0.250
**Laboratory findings**				
IL-8 admission, pg/mL	48.90 (18.50–248.30)	99.80 (37.20–512.50)	23.10 (12.30–43.80)	<0.001
sST2 admission, ng/mL	70.50 (32.20–133.50)	78.30 (44.40–159.90)	43.10 (20.60–110.80)	0.005
hs-cTnI admission, ng/L	20.0(10.0–67.0)	40.0(10.0–100.0)	10.0(10.0–30.0)	0.008
eGFR, mL/min/1.73 m²	89.50(70.20–120.80)	81.60 (66.00–110.20)	100.80 (76.80–137.60)	0.010
PaO₂/FiO₂ ratio	238.00(192.00–323.50)	222.00 (180.00–278.00)	295.00(234.00–366.5)	<0.001
WBC, ×10⁹/L	10.50(6.90–14.20)	10.90 (8.20–15.00)	8.40 (6.20–12.60)	0.018
IL-6 admission, pg/mL	62.10 (21.80–174.20)	70.50 (22.10–218.30)	57.20 (21.60–147.80)	0.198
PCT, ng/mL	0.26 (0.08–1.24)	0.32 (0.07–1.60)	0.22 (0.08–1.04)	0.745
CRP, mg/L	28.50(10.00–105.20)	32.50 (10.00–114.50)	21.00 (10.00–90.80)	0.278
D-dimer, μg/mL	0.85 (0.40–1.88)	0.94 (0.50–2.02)	0.62 (0.36–1.46)	0.098
Albumin, g/L	35.00(31.00–39.00)	34.50 (31.00–37.00)	37.00 (33.00–40.00)	0.055
BNP, pg/mL	205.60(80.20–902.30)	435.80(104.50–1180.50)	112.40(80.00–704.80)	0.052
**Severity scores**				
SOFA score	3.00 (2.00–4.00)	3.00 (2.00–4.00)	2.00 (2.00–4.00)	0.038
CURB-65 score	2.00 (1.00–3.00)	2.00 (2.00–3.00)	2.00 (1.00–2.00)	0.004
PSI score	105.00(90.00–126.00)	116.00(92.00–131.00)	96.00(81.00–111.00)	0.002
**Clinical outcomes and support**				
NIV support	82/143 (57.3)	62/86 (72.1)	20/57 (35.1)	<0.001
Prior PCI/CABG	5/146 (3.4)	5/88 (5.7)	0/58 (0.0)	0.142
Baseline antiplatelet therapy	27/146 (18.5)	21/88 (23.9)	6/58 (10.3)	0.058
Respiratory failure	88/143 (61.5)	64/86 (74.4)	24/57 (42.1)	<0.001
Heart failure	48/143 (33.6)	33/86 (38.4)	15/57 (26.3)	0.137
Pleural effusion	48/143 (33.6)	34/86 (39.5)	14/57 (24.6)	0.062
Sepsis	86/144 (59.7)	57/88 (64.8)	29/56 (51.8)	0.120
MODS	80/144 (55.6)	53/88 (60.2)	27/56 (48.2)	0.158
28-day mortality	15/146 (10.3)	15/88 (17.0)	0/58 (0.0)	0.003

Data are presented as median (interquartile range [IQR]) or n/N (%), where N represents the number of patients with available data. Denominators varied because of missing data. Data on respiratory failure, heart failure, and pleural effusion were available for 143 patients (86 with myocardial injury and 57 without), whereas data on sepsis and multiple organ dysfunction syndrome (MODS) were available for 144 patients (88 with myocardial injury and 56 without). Missing values reflected incomplete documentation during the index hospitalization rather than loss to follow-up. No imputation was performed, and available-case analysis was used. Continuous variables were compared using the Mann–Whitney U test, and categorical variables using the χ^2^ test or Fisher’s exact test, as appropriate. P values <0.05 are shown in bold. Abbreviations: SCAP, severe community-acquired pneumonia; IL-8, interleukin-8; sST2, soluble suppression of tumorigenicity 2; hs-cTnI, high-sensitivity cardiac troponin I; eGFR, estimated glomerular filtration rate; SOFA, Sequential Organ Failure Assessment; PSI, Pneumonia Severity Index; NIV, non-invasive ventilation.

### 3.2. Admission biomarker profiles according to myocardial injury status

**Figure 1.**
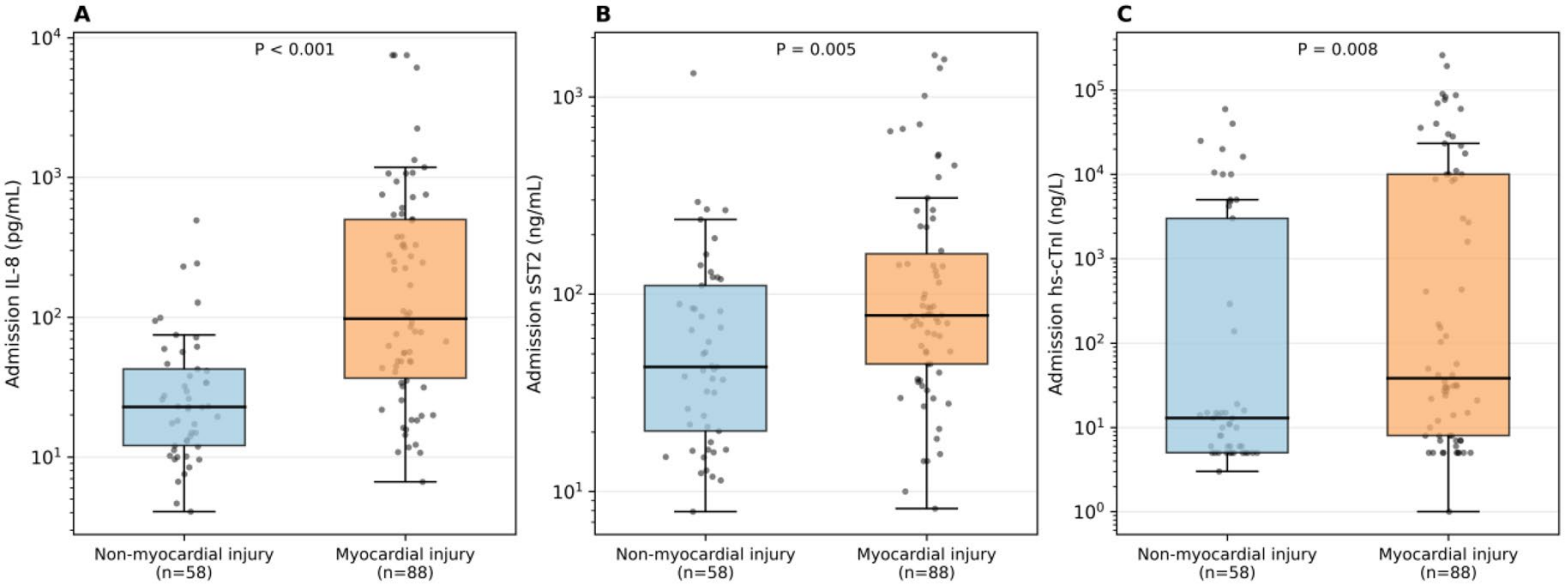
Admission biomarker distributions according to guideline-consistent myocardial injury status. Violin/box plots showing the distributions of admission IL-8, sST2, and hs-cTnI in patients with guideline-consistent myocardial injury (n = 88) and without myocardial injury (n = 58).(A) Admission IL-8.；(B) Admission sST2.；(C) Admission hs-cTnI. All comparisons were based on the available observed data (total n = 146). No statistical imputation was performed, and no imputed biomarker values were included in this figure. hs-cTnI is shown for descriptive purposes only because it contributed to the definition of the primary endpoint and was therefore excluded from the primary IL-8 + sST2 prediction model to avoid circularity. Abbreviations: hs-cTnI, high-sensitivity cardiac troponin I; IL-8, interleukin-8; sST2, soluble suppression of tumorigenicity 2.

The distributions of admission IL-8, sST2, and hs-cTnI according to guideline-consistent myocardial injury status are shown in Figure 1. Consistent with the baseline comparisons in Table 1, admission IL-8 and sST2 levels were higher in patients with myocardial injury than in those without myocardial injury, with IL-8 showing a more pronounced separation between groups than sST2. Admission hs-cTnI concentrations were also higher in the myocardial injury group; however, hs-cTnI is presented for descriptive purposes only because it contributed to the definition of the primary endpoint and was therefore not evaluated as an independent diagnostic predictor.

### 3.3. Diagnostic performance of admission IL-8, sST2, and the combined IL-8 + sST2 model

**Figure 2.**
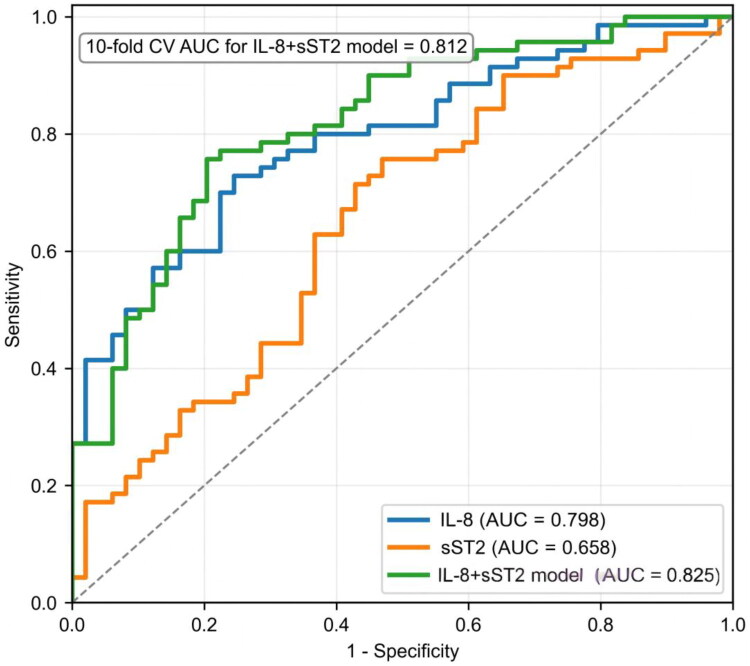
Receiver operating characteristic curves for admission IL-8, sST2, and the combined IL-8 + sST2 model. Receiver operating characteristic curves comparing admission IL-8, admission sST2, and the combined IL-8 + sST2 model for guideline-consistent myocardial injury. In the final analysis, the AUCs were 0.798 for IL-8, 0.658 for sST2, and 0.825 for the combined model. The internally cross-validated AUC for the combined model was 0.812. Calibration and decision curve analyses are presented separately in Figure 4.No external validation cohort was available. Abbreviations: AUC, area under the curve; IL-8, interleukin-8; ROC, receiver operating characteristic; sST2, soluble suppression of tumorigenicity 2.

The discriminatory performance of admission biomarkers and conventional clinical severity scores is summarized in Table 2 and Figure 2.

**Table 2.** Discriminatory performance of admission biomarkers, the IL-8 + sST2 model, and conventional severity scores for guideline-consistent myocardial injury.

Marker/model	N	AUC (95% CI)	Cut-off	Sensitivity	Specificity	Youden index	TP/TN/FP/FN
IL-8 admission	146	0.798 (0.725–0.871)	43.53	0.739	0.741	0.480	65/43/15/23
sST2 admission	146	0.658 (0.558–0.748)	44.30	0.761	0.517	0.279	67/30/28/21
IL-8 + sST2 model	146	0.825 (0.751–0.895)	0.558	0.761	0.810	0.572	67/47/11/21
SOFA score	144	0.612 (0.518–0.714)	3.00	0.663	0.569	0.232	57/33/25/29
CURB-65 score	145	0.648 (0.550–0.735)	3.00	0.379	0.828	0.207	33/48/10/54
PSI score	144	0.668 (0.568–0.765)	115.00	0.511	0.786	0.297	45/44/12/43

Table Note: the IL-8 + sST2 model was internally assessed using repeated 10-fold cross-validation, yielding a mean cross-validated AUC of 0.812 (95% CI: 0.742–0.878). Pairwise AUC comparisons were performed using the DeLong test with bonferroni correction. The corrected significance threshold was *p* < 0.0083. The IL-8 + sST2 model showed higher AUC than SOFA (*p* = 0.002), CURB-65 (*p* = 0.008), and PSI (*p* = 0.004). The observed AUC increase of the combined model over IL-8 alone was modest.

Abbreviations: AUC, area under the receiver operating characteristic curve; CI, confidence interval; FP, false positive; FN, false negative; IL-8, interleukin-8; PSI, Pneumonia Severity Index; SOFA, Sequential Organ Failure Assessment; sST2, soluble suppression of tumorigenicity 2; TN, true negative; TP, true positive.

Admission IL-8 had an AUC of 0.798 (95% CI: 0.725–0.871). At the optimal cut-off value of 43.53 pg/mL, IL-8 yielded a sensitivity of 73.9% and a specificity of 74.1%.

Admission sST2 had an AUC of 0.658 (95% CI: 0.558–0.748). At the optimal cut-off value of 44.30 ng/mL, sST2 yielded a sensitivity of 76.1% and a specificity of 51.7%.

The combined IL-8 + sST2 model showed the highest discriminatory performance among the evaluated biomarker models, with an AUC of 0.825 (95% CI: 0.751–0.895). At the optimal probability threshold, the model achieved a sensitivity of 76.1% and a specificity of 81.0%. Internal validation using repeated 10-fold cross-validation yielded a mean cross-validated AUC of 0.812 (95% CI: 0.742–0.878). Model calibration and clinical utility were evaluated separately and are presented in the subsequent section.

At the selected threshold, 21 of 88 patients with myocardial injury were not identified by the combined model.

The AUCs of SOFA, CURB-65, and PSI were 0.612, 0.648, and 0.668, respectively. In pairwise DeLong comparisons with Bonferroni correction, the IL-8 + sST2 model showed significantly higher discrimination than SOFA (*p* = 0.002), CURB-65 (*p* = 0.008), and PSI (*p* = 0.004).

### 3.4. Univariable associations with guideline-consistent myocardial injury

Univariable logistic regression results are shown in Table 3. Admission IL-8 was associated with guideline-consistent myocardial injury, with an OR of 1.079 per 10 pg/mL increase (95% CI: 1.030–1.132; *p* = 0.001).

**Table 3.** Logistic regression analysis of variables associated with guideline-consistent myocardial injury.

Predictor	Unit	N	OR	95% CI	P value
IL-8 admission	per 10 pg/mL	146	1.079	1.030–1.132	0.001
sST2 admission	per 10 ng/mL	146	1.019	0.998–1.041	0.068
Egfr	per 10 mL/min/1.73 m²	146	0.865	0.792–0.948	0.002
SOFA score	per 1 point	144	1.218	1.024–1.448	0.028
CURB-65 score	per 1 point	145	1.758	1.158–2.668	0.008
PSI score	per 10 points	144	1.148	1.038–1.269	0.006

Table note: Odds ratios were estimated from univariable logistic regression models and should be interpreted as associations rather than causal effects. hs-cTnI was not included because it contributed to the definition of guideline-consistent myocardial injury.

Abbreviations: CI, confidence interval; eGFR, estimated glomerular filtration rate; IL-8, interleukin-8; OR, odds ratio; PSI, Pneumonia Severity Index; SOFA, Sequential Organ Failure Assessment; sST2, soluble suppression of tumorigenicity 2.

Admission sST2 showed a weaker association that did not reach conventional statistical significance (OR per 10 ng/mL: 1.019, 95% CI: 0.998–1.041; *p* = 0.068).

Higher eGFR was associated with lower odds of myocardial injury (OR per 10 mL/min/1.73 m^2^: 0.865, 95% CI: 0.792–0.948; *p* = 0.002). SOFA, CURB-65, and PSI scores were also associated with guideline-consistent myocardial injury in univariable analyses.

### 3.5. Exploratory analysis: eGFR-stratified hs-cTnI interpretation

In an exploratory analysis, we compared the manufacturer-provided overall 99th-percentile URL of 26.4 ng/L with eGFR-stratified hs-cTnI thresholds. The stratified thresholds were 26.4 ng/L for eGFR ≥60 mL/min/1.73 m^2^, 39.6 ng/L for eGFR 30–<60 mL/min/1.73 m^2^, and 66.0 ng/L for eGFR <30 mL/min/1.73 m^2^, using the proportional adjustment approach derived from Chen et al. Results are presented in Table 4.

**Table 4.** Exploratory analysis: eGFR-stratified hs-cTnI interpretation.

eGFR stratum (mL/min/1.73 m²)	N	Overall URL (26.4 ng/L): elevated, n	Overall URL: discordant elevation, n (%)	eGFR-stratified threshold, ng/L	eGFR-stratified threshold: elevated, n	eGFR-stratified threshold: discordant elevation, n (%)	Discordant elevation reduction, n
≥60	128	74	19/74 (25.7)	26.4	74	19/74 (25.7)	0
30–<60	13	7	0/7 (0.0)	39.6	5	0/5 (0.0)	0
<30	5	5	1/5 (20.0)	66.0	5	1/5 (20.0)	0
Total	146	86	20/86 (23.3)	—	84	20/84 (23.8)	0

Table note: Discordant elevation was defined as hs-cTnI elevation according to the specified threshold in patients who did not meet the final guideline-consistent myocardial injury classification (based on sex-specific URLs: 33.5 ng/L for males and 19.8 ng/L for females). Because only 18 patients had eGFR <60 mL/min/1.73 m**^2^**, this analysis was considered exploratory. Although eGFR-stratified thresholds reduced the total number of hs-cTnI elevations from 86 to 84, the reclassified patients were already classified as having guideline-consistent myocardial injury based on sex-specific URLs; therefore, no discordant hs-cTnI elevations were eliminated.

Using the overall URL of 26.4 ng/L, 86 patients were classified as having hs-cTnI elevation. Among them, 20 patients did not meet the final guideline-consistent myocardial injury classification (based on sex-specific URLs) and were therefore considered to have discordant hs-cTnI elevation. When eGFR-stratified thresholds were applied, 84 patients were classified as having hs-cTnI elevation, with 20 discordant elevations.

In patients with eGFR ≥60 mL/min/1.73 m^2^ (*n* = 128), the overall URL and eGFR-stratified thresholds were identical (26.4 ng/L); therefore, the number of hs-cTnI elevations and discordant elevations did not differ between approaches.

Among patients with eGFR 30–<60 mL/min/1.73 m^2^ (*n* = 13), the stratified threshold of 39.6 ng/L reduced the number of hs-cTnI elevations from 7 (using 26.4 ng/L) to 5, but no discordant elevations were observed under either threshold.

Among patients with eGFR <30 mL/min/1.73 m^2^ (*n* = 5), both the overall URL (26.4 ng/L) and the stratified threshold (66.0 ng/L) identified 5 hs-cTnI elevations, including 1 discordant elevation.

Overall, eGFR-stratified thresholds reduced the number of patients classified as having hs-cTnI elevation from 86 to 84 but did not reduce the number of discordant hs-cTnI elevations in this cohort. Because only 18 patients had eGFR <60 mL/min/1.73 m^2^, this analysis was exploratory and not suitable for definitive inference regarding renal function-adjusted hs-cTnI thresholds.

### 3.6. Exploratory subgroup analysis: patients with CURB-65 ≤ 1

As shown in Table 5, Among 38 patients with CURB-65 ≤ 1, 21 patients (55.3%) had guideline-consistent myocardial injury. Using the overall cohort-derived IL-8 cut-off of 43.53 pg/mL, 17 patients were classified as IL-8 low and 21 as IL-8 high.

**Table 5.** Exploratory subgroup analysis among patients with CURB-65 ≤ 1.

Variable	IL-8 ≤ 43.53 pg/mL (*n* = 17)	IL-8 > 43.53 pg/mL (*n* = 21)	P value	sST2 ≤ 44.30 ng/mL (*n* = 17)	sST2 > 44.30 ng/mL (*n* = 21)	P value
Myocardial injury, n (%)	5 (29.4)	16 (76.2)	0.004	8 (47.1)	13 (61.9)	0.368
Elevated hs-cTnI, n (%)	6 (35.3)	14 (66.7)	0.056	8 (47.1)	12 (57.1)	0.542
SOFA ≥3, n (%)	7 (41.2)	11 (52.4)	0.493	8 (47.1)	10 (47.6)	0.974
PSI class IV–V, n (%)	7 (41.2)	13 (61.9)	0.205	9 (52.9)	11 (52.4)	0.974

Table note: Patients were classified as low risk according to CURB-65 ≤ 1. Biomarker groups were defined using the optimal cut-offs derived from the overall cohort. Interaction tests did not show significant effect modification by CURB-65 subgroup status: IL-8 × subgroup, *p* = 0.182; sST2 × subgroup, *p* = 0.294. This subgroup analysis was exploratory. Elevated hs-cTnI was defined using the manufacturer-provided overall upper reference limit of 26.4 ng/L. Abbreviations: hs-cTnI, high-sensitivity cardiac troponin I; IL-8, interleukin-8; PSI, Pneumonia Severity Index; SOFA, Sequential Organ Failure Assessment; sST2, soluble suppression of tumorigenicity 2.

In the IL-8 high group, myocardial injury was observed in 16 of 21 patients (76.2%), compared with 5 of 17 patients (29.4%) in the IL-8 low group (*p* = 0.004). The interaction test did not show significant effect modification by CURB-65 subgroup status (P for interaction = 0.182).

Using the overall cohort-derived sST2 cut-off of 44.30 ng/mL, myocardial injury occurred in 13 of 21 patients (61.9%) in the sST2 high group and 8 of 17 patients (47.1%) in the sST2 low group (*p* = 0.368). The interaction test for sST2 was also non-significant (P for interaction = 0.294).

Within the CURB-65 ≤ 1 subgroup, myocardial injury prevalence differed between the IL-8 strata, whereas no statistically significant difference was observed between the sST2 strata. Interaction tests were not statistically significant. Because of the small subgroup size, these findings were considered exploratory.

### 3.7. Exploratory simplified IL-8 + sST2 score

**Figure 3.**
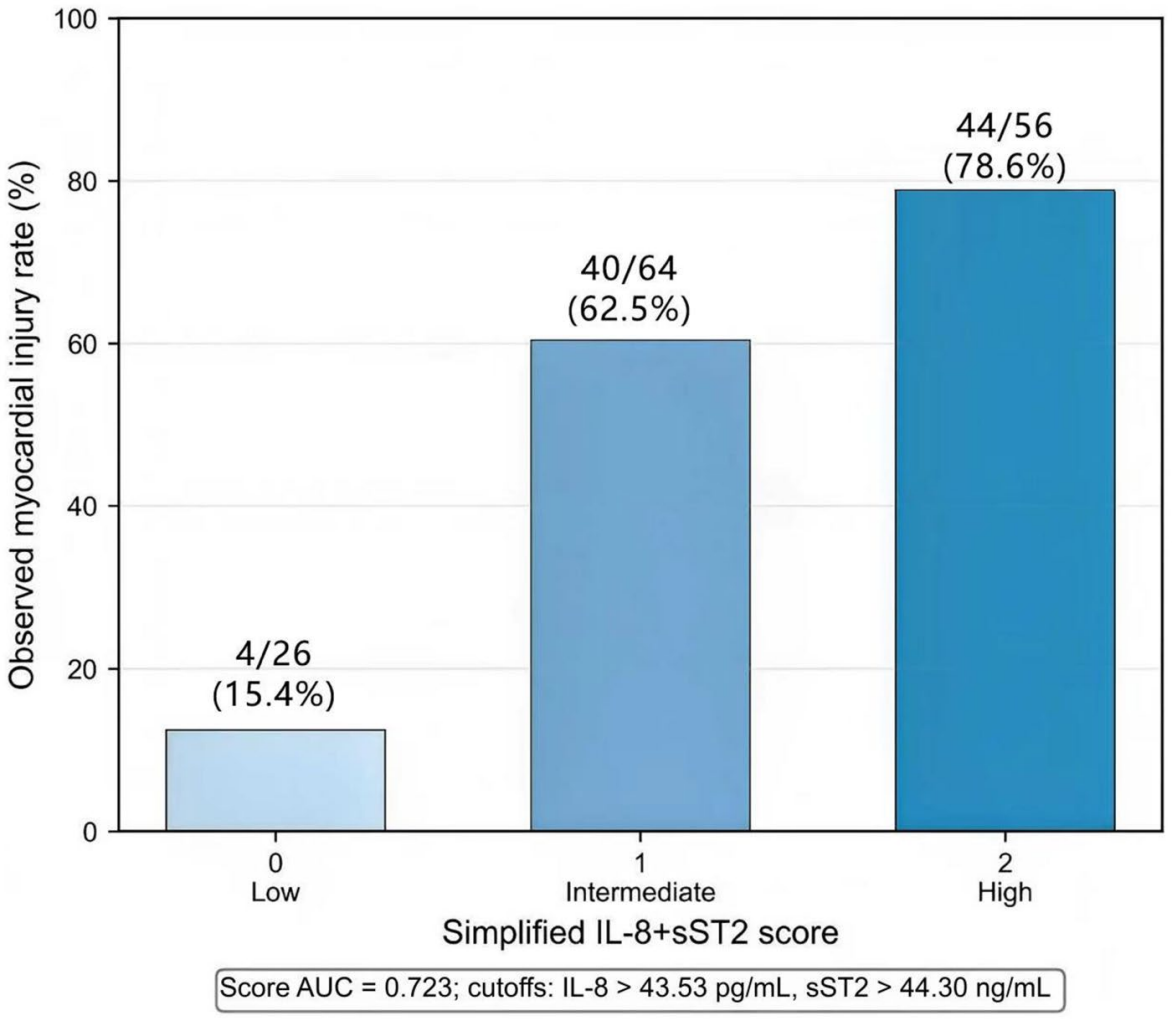
Prevalence of guideline-consistent myocardial injury across simplified IL-8 + sST2 score categories. Bar plot showing the prevalence of guideline-consistent myocardial injury across simplified IL-8 + sST2 score categories. Myocardial injury occurred in 15.4% of patients with a score of 0, 62.5% with a score of 1, and 78.6% with a score of 2, with a significant increasing trend across score categories (P for trend <0.001). Abbreviations: IL-8, interleukin-8; sST2, soluble suppression of tumorigenicity 2.

To facilitate bedside risk stratification, an exploratory simplified IL-8 + sST2 score was constructed and is presented in Table 6 and Figure 3. One point was assigned for IL-8 > 43.53 pg/mL and one point for sST2 > 44.30 ng/mL, resulting in a total score ranging from 0 to 2.

**Table 6.** Exploratory simplified IL-8 + sST2 score for guideline-consistent myocardial injury.

Panel A. Myocardial injury prevalence across score categories				
Score	N	Myocardial injury, n	No myocardial injury, n	Myocardial injury, n(%)
0	26	4	22	15.4%
1	64	40	24	62.5%
2	56	44	12	78.6%

**Table:** 

Panel B. Diagnostic performance at different score thresholds								
Threshold	TP	TN	FP	FN	Sensitivity	Specificity	PPV	NPV
≥1	84	22	36	4	0.955	0.379	0.700	0.846
≥2	44	46	12	44	0.500	0.793	0.786	0.511

Table note: The simplified score was calculated by assigning 1 point for IL-8 > 43.53 pg/mL and 1 point for sST2 > 44.30 ng/mL. The total score ranged from 0 to 2. The AUC of the simplified score was 0.723 (95% CI: 0.643–0.803), and the 10-fold cross-validated AUC was 0.758. A significant increasing trend in myocardial injury prevalence across score categories was confirmed by the Cochran–Armitage trend test (*Z* = 5.14, χ^2^ = 26.46, P for trend = 2.70 × 10^−7^). This score was exploratory and requires external validation.

Abbreviations: AUC, area under the receiver operating characteristic curve; FN, false negative; FP, false positive; IL-8, interleukin-8; NPV, negative predictive value; PPV, positive predictive value; sST2, soluble suppression of tumorigenicity 2; TN, true negative; TP, true positive.

The prevalence of guideline-consistent myocardial injury increased stepwise across score categories. Myocardial injury occurred in 4 of 26 patients with a score of 0 (15.4%), 40 of 64 patients with a score of 1 (62.5%), and 44 of 56 patients with a score of 2 (78.6%). The increasing trend across score categories was statistically significant (P for trend <0.001).

The simplified score had an AUC of 0.723 (95% CI: 0.643–0.803), with a 10-fold cross-validated AUC of 0.758. At a threshold of ≥1 point, the score achieved high sensitivity of 95.5% and a negative predictive value of 84.6%, although specificity was limited at 37.9%. At a threshold of 2 points, specificity improved to 79.3%, with a positive predictive value of 78.6%.

### 3.8. Calibration and decision curve analysis

**Figure 4.**
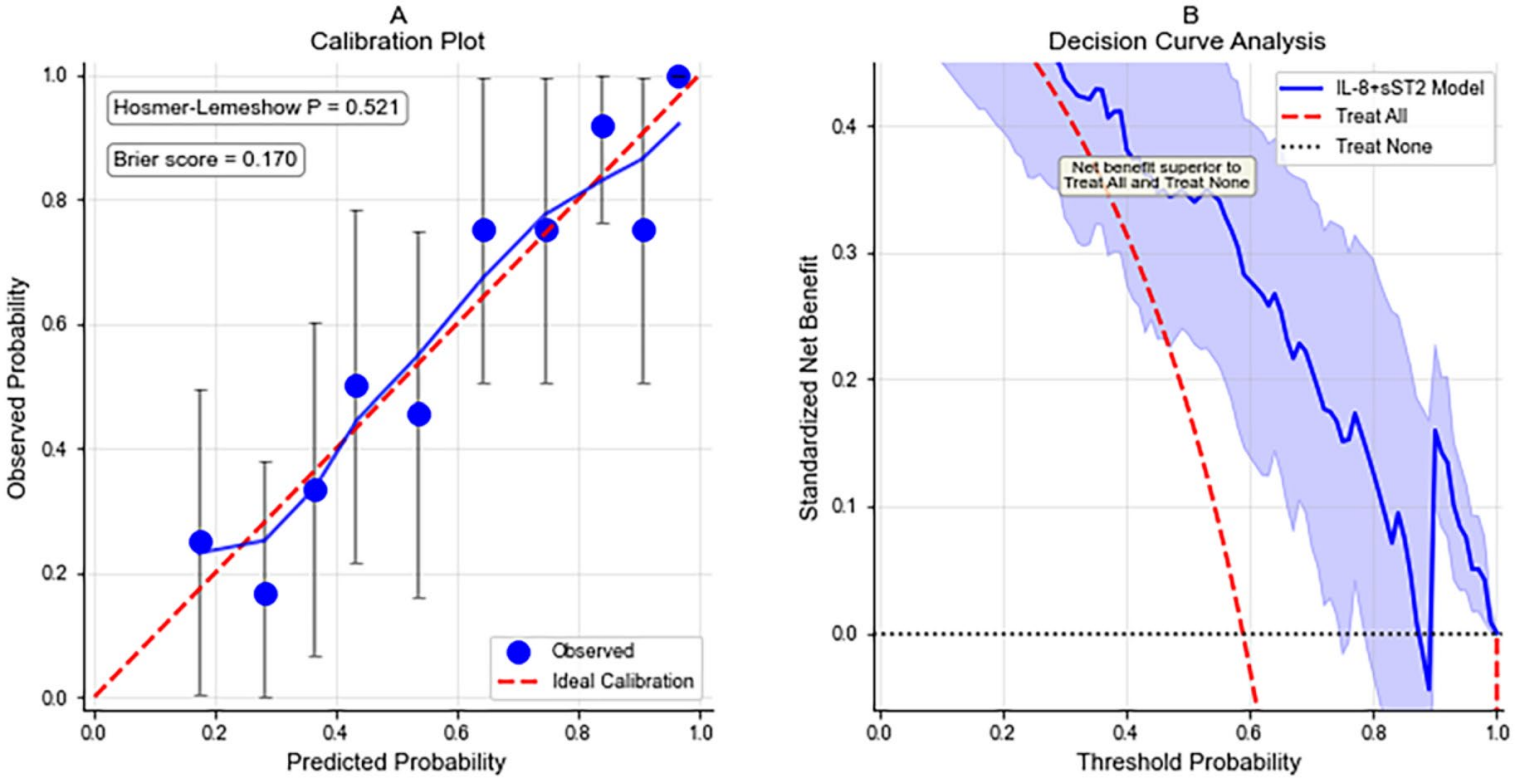
Calibration and decision curve analysis of the combined IL-8 + sST2 model. (A) Calibration plot of the combined IL-8 + sST2 model for predicting guideline-consistent myocardial injury. (B) Decision curve analysis of the combined IL-8 + sST2 model compared with the treat-all and treat-none strategies. Abbreviations: DCA, decision curve analysis.

Calibration and decision curve analyses of the combined IL-8 + sST2 model are shown in Figure 4.

Calibration analysis showed agreement between predicted and observed probabilities of guideline-consistent myocardial injury. The Hosmer–Lemeshow goodness-of-fit test was non-significant (*p* = 0.521), and the Brier score was 0.170.

Decision curve analysis is presented in Figure 4(B). Across the evaluated threshold probabilities, the combined IL-8 + sST2 model showed a higher net benefit than the treat-all and treat-none strategies.

## 4. Discussion

In this prospective cohort of 146 patients with severe community-acquired pneumonia, admission IL-8 and sST2 were evaluated as candidate biomarkers for early risk stratification of guideline-consistent myocardial injury. IL-8 showed the stronger individual association and discrimination, whereas sST2 had weaker standalone performance. The combined model achieved an apparent AUC of 0.825 and an internally cross-validated AUC of 0.812. Although discrimination was numerically higher than that of IL-8 alone, the incremental improvement was modest and the model missed a substantial proportion of myocardial injury cases. Therefore, these findings support a potential adjunctive role for IL-8 and sST2 rather than clinically actionable diagnostic performance.

The association between IL-8 and myocardial injury is biologically plausible. IL-8 is a neutrophil-recruiting chemokine that amplifies systemic inflammation, activates endothelium, and promotes microvascular dysfunction [28]. In severe pneumonia, elevated IL-8 reflects inflammatory burden and microvascular stress, both of which are recognized contributors to myocardial injury [29,30]. In this cohort, IL-8 demonstrated greater discriminatory ability (AUC 0.798) than sST2 and conventional severity scores, and remained associated with myocardial injury in univariable analysis. These data support the hypothesis that acute inflammatory activation—quantified by IL-8—is closely linked to troponin-defined myocardial injury in SCAP. However, given the observational design, IL-8 should be interpreted as a biomarker of inflammatory risk and disease activity rather than as evidence of a direct causal mechanism [31].

In contrast, sST2 showed more limited discrimination in this cohort (AUC 0.658; univariable OR per 10 ng/mL: 1.019, *p* = 0.068). Several explanations may account for this. First, sST2 is predominantly a marker of myocardial strain and fibrosis in chronic heart failure [32], whereas SCAP-associated myocardial injury is driven by acute inflammation and oxygen supply–demand mismatch [33]. Second, sST2 levels in acute settings may be influenced by renal function and baseline cardiac status, which were not fully adjusted for in this exploratory analysis. Third, the lack of statistical significance may reflect limited sample size (type II error) rather than true absence of association [34]. The combined IL-8 + sST2 model showed numerically higher discrimination than IL-8 alone (AUC 0.825 vs 0.798), but this modest improvement warrants cautious interpretation. Without a formal test of incremental value and external validation, the added contribution of sST2 cannot be confirmed [35].

A notable finding was the absence of a statistically significant association between prior coronary artery disease and admission myocardial injury, together with a higher prevalence of respiratory failure among patients with myocardial injury. These observations raise the possibility that systemic inflammation, endothelial dysfunction, hypoxemia, and myocardial oxygen supply–demand imbalance contribute to myocardial injury in SCAP. Such mechanisms would be compatible with type 2 myocardial infarction, sepsis-related myocardial dysfunction, or other forms of non-ischemic myocardial injury [3,36,37]. However, these observational and largely unadjusted findings do not establish a specific mechanism and do not exclude plaque-related myocardial ischemia. Because serial hs-cTnI measurements, electrocardiographic findings, and cardiac imaging were not systematically available for mechanistic adjudication, the relative contributions of these pathways could not be determined.

Notably, all 15 deaths within 28 days occurred among patients with admission myocardial injury (17.0% vs. 0.0%, *p* = 0.003), supporting the prognostic relevance of hs-cTnI in this population and aligning with previous reports [9,38]. However, the present study was not designed to determine whether myocardial injury contributed causally to mortality or primarily served as a marker of greater disease severity and multi-organ dysfunction. Severe inflammation, respiratory failure, tissue hypoxia, and myocardial injury may represent interrelated components of critical illness, and the direction of these relationships cannot be established from this observational analysis. Mechanistic studies and appropriately designed longitudinal investigations are needed to distinguish whether myocardial injury is a mediator of poor outcomes, a marker of the severity of the underlying illness, or both.

In the exploratory subgroup of patients with CURB-65 ≤ 1 (*n* = 38), the prevalence of myocardial injury was 55.3%, highlighting that traditional severity scores—developed to predict mortality—do not capture cardiac complications. The IL-8 association was directionally consistent with the main analysis: myocardial injury occurred in 16/21 (76.2%) of IL-8-high versus 5/17 (29.4%) of IL-8-low patients. This finding suggests that even among patients with low clinical severity scores, IL-8 may identify those with heightened inflammatory burden and correspondingly higher likelihood of myocardial injury. However, the interaction term between IL-8 and CURB-65 subgroup was not statistically significant (*p* = 0.182), indicating no evidence that the IL-8 effect is modified by CURB-65 stratum. Rather than supporting a unique effect in low-risk patients, this observation suggests that IL-8 provides risk information that is independent of CURB-65 stratification [24,38,39]. These findings indicate that IL-8 may provide complementary information for early cardiac risk assessment in SCAP, particularly among patients with lower CURB-65 scores. However, IL-8 cannot replace hs-cTnI-based assessment, as the model missed 21 of 88 myocardial injury cases overall. The simplified IL-8 + sST2 score—showing stepwise prevalence increases from 15.4% to 78.6%—was derived with data-driven cut-offs and requires independent validation before clinical use.

The exploratory eGFR-stratified hs-cTnI analysis did not show a meaningful reduction in discordant hs-cTnI elevations compared with the conventional fixed threshold. This finding likely reflects the small number of patients with moderate-to-severe renal dysfunction in the expanded cohort: only 18 patients had eGFR <60 mL/min/1.73 m^2^. Therefore, the present data are insufficient to determine whether renal-function-adjusted hs-cTnI thresholds are useful in SCAP [12]. Larger cohorts enriched for renal impairment would be required to evaluate this question more rigorously.

The comparison with conventional severity scores also deserves cautious interpretation. SOFA, CURB-65, and PSI were designed to assess illness severity and prognosis rather than myocardial injury [40]. Their lower AUCs do not establish that the biomarker model should replace these scores. At the selected model threshold, 21 of 88 patients with myocardial injury were missed, indicating that IL-8 and sST2 cannot be used to rule out myocardial injury or replace serial hs-cTnI measurement, electrocardiography, or clinically indicated cardiac imaging [41].

Several limitations should be acknowledged. First, this was a single-center study without an external validation cohort. Although the combined IL-8 + sST2 model was internally assessed using repeated cross-validation, the estimated performance may remain optimistic. In addition, the simplified score and its biomarker cut-offs were derived from the same cohort. Therefore, the model, score, and cut-offs require validation in independent, preferably multicenter cohorts before clinical implementation. Prospective studies incorporating repeated measurements of IL-8, sST2, and hs-cTnI are also needed to evaluate biomarker changes over time and their relationship with incident myocardial injury and clinical outcomes.

Second, myocardial injury status was determined primarily using a single hs-cTnI measurement obtained at admission. Of the 146 patients, 88 met the prespecified criteria for myocardial injury at admission, whereas 58 did not. Thus, the present findings concern the association of admission IL-8 and sST2 with myocardial injury status at hospital presentation rather than their ability to predict myocardial injury developing subsequently. Because the study did not include a prespecified protocol for systematic serial hs-cTnI measurement or adjudication of subsequent clinical acute myocardial infarction, incident myocardial injury or AMI events occurring after admission were not systematically captured. Therefore, the ability of admission IL-8 or sST2 to discriminate subsequent AMI events could not be evaluated in this cohort. Serial hs-cTnI data were also unavailable for all patients, preventing consistent differentiation between acute and chronic myocardial injury. Although patients with confirmed type 1 acute coronary syndromes were excluded, type 2 myocardial infarction and non-ischemic myocardial injury could not be systematically distinguished because complete serial troponin, electrocardiographic, and cardiac imaging data were unavailable for mechanistic adjudication.

Third, the analyses were based mainly on univariable associations, and residual confounding by illness severity, renal function, respiratory failure, and pre-existing cardiovascular disease cannot be excluded. The study also did not include a standardized prospective assessment of baseline ischemic cardiovascular risk using a validated risk prediction tool. Although major cardiovascular comorbidities, prior coronary revascularization, and baseline antiplatelet therapy were recorded, we could not determine whether the associations of IL-8 and sST2 with myocardial injury differed according to underlying cardiovascular risk. Larger prospective studies incorporating multivariable adjustment and standardized cardiovascular risk assessment are warranted.

Fourth, only 18 patients had an eGFR below 60 mL/min/1.73 m^2^, limiting evaluation of kidney function-specific hs-cTnI thresholds and precluding stable estimation of an eGFR-by-hs-cTnI interaction. Because most patients had preserved renal function, the present study cannot establish whether IL-8 or sST2 provides clinically meaningful additional information beyond guideline-recommended hs-cTnI interpretation in patients with substantial renal impairment.

Finally, this study did not assess whether biomarker-guided testing changes clinical management, healthcare utilization, or patient outcomes, and it was not designed to analyse mortality timing or other time-to-event outcomes. Accordingly, the findings should be considered preliminary and hypothesis-generating. Whether repeated or biomarker-guided assessment improves clinically meaningful outcomes should be evaluated in dedicated prospective studies.

## 5. Conclusion

Admission IL-8 was associated with guideline-consistent myocardial injury in patients with severe community-acquired pneumonia, whereas sST2 showed weaker individual discrimination. The combined IL-8 + sST2 model demonstrated numerically higher discrimination than IL-8 alone; however, the incremental value of sST2 and the clinical utility of the simplified biomarker score were not established. These biomarkers should therefore be considered potential adjuncts rather than alternatives to hs-cTnI-based assessment. Given the single-centre, hypothesis-generating nature of this study, external validation in independent cohorts is required before IL-8, sST2, or the simplified score can be recommended for clinical use.

## Data Availability

The datasets generated and analyzed during the current study are available from the corresponding author upon reasonable request.
